# The complete chloroplast genome of the extremely drought-resistant plant *Primulina ophiopogoides*

**DOI:** 10.1080/23802359.2020.1768950

**Published:** 2020-06-02

**Authors:** Yongbin Lu, Xinmei Qin, Zhangping Huang, Qiang Zhang, Lina Shen

**Affiliations:** a Guangxi Key Laboratory of Plant Conservation and Restoration Ecology in Karst Terrain, Guangxi Institute of Botany, Guilin, Guangxi, China; b Key Laboratory of Karst Ecosystem and Treatment of Rocky Desertification, Ministry of Land and Resources, Institute of Karst Geology of Chinese Academy of Geology Sciences, Guilin, Guangxi, China

**Keywords:** *Primulina ophiopogoides*, chloroplast genome, phylogenetics

## Abstract

*Primulina ophiopogoides* is a perennial herb of Gesneriaceae distributed on the limestone rocks. Here, the complete chloroplast (cp) genome of *P. ophiopogoides* was assembled and characterized. The cp genome is in a total length of 152,718 bp with the typical quadripartite structure, containing 2 inverted repeats (IRs) of 25,472 bp separated by a large single-copy (LSC) region of 83,615 bp and a small single-copy (SSC) region of 18,159 bp. The whole cp genome of *P. ophiopogoides* contains 131 genes, including 86 protein-coding genes, 37 tRNAs genes, and 8 rRNAs. The phylogenetic analysis indicated that *P. ophiopogoides* displayed a closer kinship to *Primulina linearifolia*.

*Primulina ophiopogoides* (D. Fang & W. T. Wang) Yin Z. Wang is a perennial herb of Gesneriaceae. It is an endemic endangered species distributed on limestone rocks in Fusui and Longzhou County, Guangxi, China, usually growing on bare and unshaded rock crevices and showing very strong tolerance to extremely drought and high temperature in summer and autumn. The rhizome of *P. ophiopogoides* is a traditional Chinese medicine mainly used for rheumatism (Editorial Committee of the Flora of China 1990; Li and Wang 2004) and it is also an ornamental plant with fleshy leaves and purplish flowers. We report the complete chloroplast (cp) genome of *P. ophiopogoides*, which can be used to test the phylogenetic relationship to its congeners and provide basic data for future analysis of the adaptation mechanism to the harsh habitats.

Total DNA was extracted from the silica-dried leaves of *P. ophiopogoides* using a modified CTAB method (Doyle 1987), which were collected from Fusui county, Guangxi, China (22°39′41′′ N, 107°55′38′′ E). The voucher specimen (ZQ20180801) was deposited at IBK. The paired-end (PE150) sequencing was performed on NovaSeq 6000 system (in Novogene corp., Tianjin, China). Approximately 2.9 GB high-quanlity clean reads were obtained after filtering. Assembly and annotation were employed with SPAdes 3.11.0 (Bankevich et al. 2012) and PGA (Qu et al. 2019). The validated complete cp genome sequence of *P. ophiopogoides* was submitted to the GenBank (accession number: MT409622).

The cp genome of *P. ophiopogoides* is 152,718 bp in length with an average GC content of 37.6%, containing one large single-copy (LSC) region of 83,615 bp and one small single-copy (SSC) region of 18,159 bp, which are separated by two inverted repeat (IR) regions of 25,472 bp. A total of 131 unique genes are encoded for the cp genome, including 86 protein-coding genes, eight ribosomal RNA genes and 37 transfer RNA genes. Among them, 15 genes (i.e., trnK-UUU, *rps*16, trnG-UCC, *atp*F, *rpo*C1, trnL-UAA, trnV-UAC, *pet*B, *pet*D, *rpl*16, *rpl*2, *ndh*B, trnI-GAU, trnA-UGC, *ndh*A) contain one intron, while *clp*P and *ycf*3 harbor two introns, and *rps*12 has trans-splicing.

To investigate the phylogenetic relationships with the closely related species, the maximum likelihood (ML) phylogenetic tree was constructed by RAxML (Stamatakis 2006) based on 12 complete cp genomes in Gesneriaceae (Figure 1). The tree shows *P. ophiopogoides* is a closer relative to *Primulina linearifolia*, which are supported by the similarity in morphological characters, such as narrow long leaves. The complete cp genome sequence of *P. ophiopogoides* can benefit further studies, such as on its adaptation mechanism.

**Figure 1.**
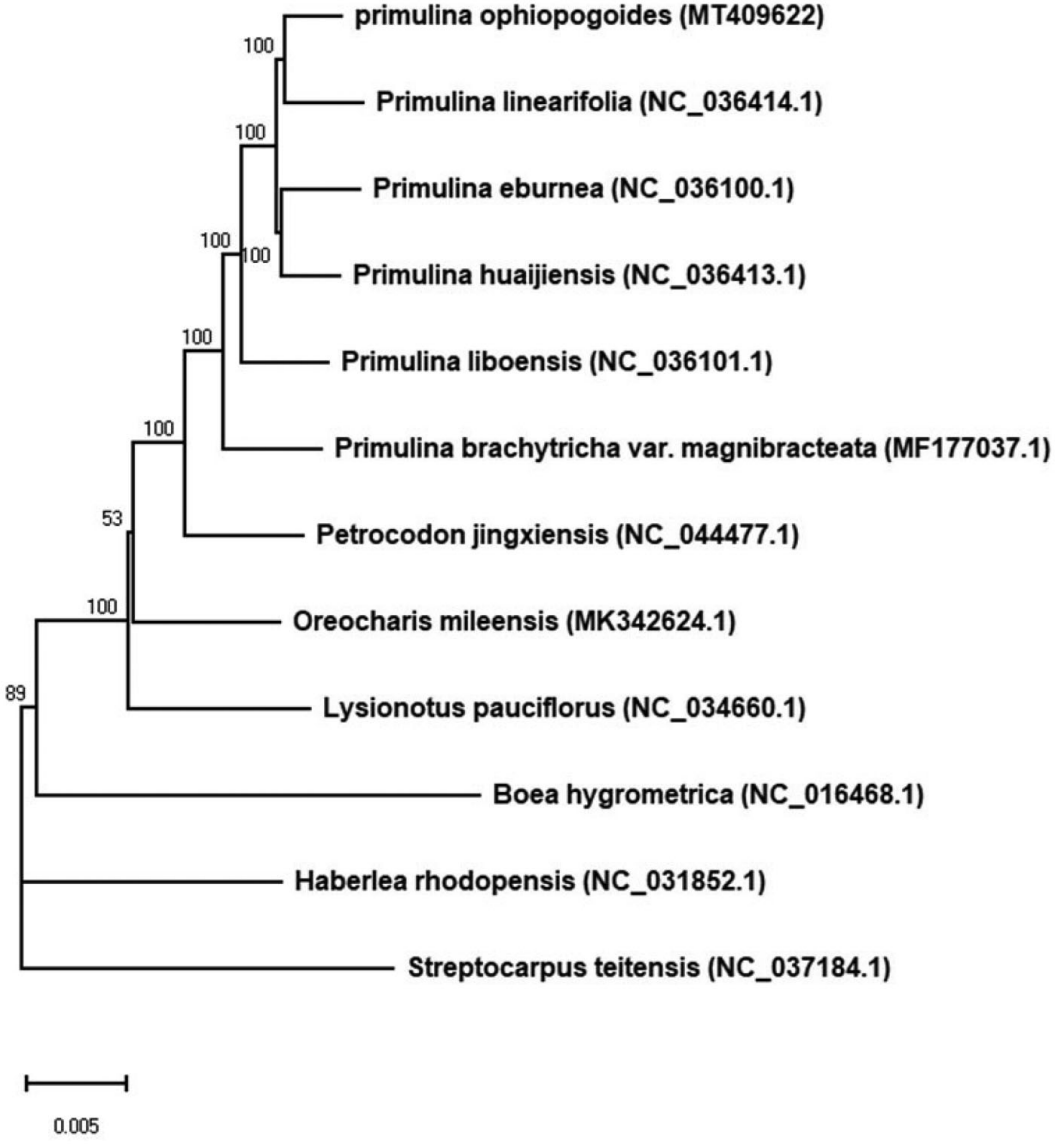
The ML tree of Gesneriaceae based on 12 complete chloroplast genome sequences. Numbers at nodes are bootstrap percentages (1000 replicates). GenBank accession numbers are shown in the figure.

## Data Availability

The data that support the findings of this study have already uploaded to the NCBI (https://www.ncbi.nlm.nih.gov/) and get the reference number (MT409622). We have permitted the data can be released before the publication of the article, but until now the data has not been released in NCBI.
